# Human Dendritic Cells: Ontogeny and Their Subsets in Health and Disease

**DOI:** 10.3390/medsci6040088

**Published:** 2018-10-08

**Authors:** Sandra Georgina Solano-Gálvez, Sonia Margarita Tovar-Torres, María Sofía Tron-Gómez, Ariane Estrella Weiser-Smeke, Diego Abelardo Álvarez-Hernández, Giorgio Alberto Franyuti-Kelly, Mijail Tapia-Moreno, Antonio Ibarra, Laila Gutiérrez-Kobeh, Rosalino Vázquez-López

**Affiliations:** 1 Departamento de Microbiología y Parasitología, Facultad de Medicina, Universidad Nacional Autónoma de México, Ciudad de México 04510, Mexico; solano-sandra@hotmail.com; 2 Departamento de Microbiología, Centro de Investigación en Ciencias de la Salud, Facultad de Ciencias de la Salud (CICSA), Universidad Anáhuac México Campus Norte, Estado de México 52786, Mexico; soniatovart@gmail.com (S.M.T.-T.); sofia.tron@hotmail.com (M.S.T.-G.); aweisers@gmail.com (A.E.W.-S.); diego.alvarez.hernandez@hotmail.com (D.A.Á.-H.); 3 Medical IMPACT, Infectious Diseases Department, Mexico City 53900, Mexico; giorgio.franyuti@gmail.com (G.A.F.-K.); tapkov@hotmail.com (M.T.-M.); 4 Coordinación del Centro de Investigación en Ciencias de la Salud, Facultad de Ciencias de la Salud (CICSA), Universidad Anáhuac México Campus Norte, Estado de México 52786, Mexico; jose.ibarra@anahuac.mx; 5 Unidad de Investigación UNAM-INC, División Investigación, Facultad de Medicina, Universidad Nacional Autónoma de México-Instituto Nacional de Cardiología “Ignacio Chávez”, Mexico City 14080, Mexico; lgutierr@unam.mx

**Keywords:** conventional DCs (cDCs), human dendritic cells, monocyte-derived DCs (moDCs), myeloid DCs (mDCs), ontogeny, plasmacytoid DCs (pDCs), subsets

## Abstract

Dendritic cells (DCs) are a type of cells derived from bone marrow that represent 1% or less of the total hematopoietic cells of any lymphoid organ or of the total cell count of the blood or epithelia. Dendritic cells comprise a heterogeneous population of cells localized in different tissues where they act as sentinels continuously capturing antigens to present them to T cells. Dendritic cells are uniquely capable of attracting and activating naïve CD4^+^ and CD8^+^ T cells to initiate and modulate primary immune responses. They have the ability to coordinate tolerance or immunity depending on their activation status, which is why they are also considered as the orchestrating cells of the immune response. The purpose of this review is to provide a general overview of the current knowledge on ontogeny and subsets of human dendritic cells as well as their function and different biological roles.

## 1. Background

Dendritic cells (DCs) were initially described in 1868 by Paul Langerhans who identified a population of cells in the skin that presented projections similar to the dendrites of neurons [1]. Almost 100 years later, in 1973, Steinman and Cohn described a cell population present in the spleen of mice and similar to those described by Langerhans. These cells showed different cellular appearance and behavior as compared to monocytes and macrophages (MΦ) and, therefore, were called DCs [2]. It was observed that this new cell population had a great capacity to initiate and modulate the immune response [3,4] and that it expressed high levels of major histocompatibility complex class II (MHC-II) and integrin alpha X (complement component 3 receptor 4 subunit) [5,6]. Subsequent studies showed that DCs do not possess CD3, CD19, or CD56 markers, which belong to other cell lineages (T and B lymphocytes and natural killer (NK) cells, respectively). Due to this, they were denominated as negative lineage cells [7]. Currently, DCs are recognized as a heterogeneous cell population whose members differ in ontogeny, anatomical location, migration, cytokine secretion pattern, and immunological functions [8]. They are located in non-lymphoid tissues where they sense (through their pattern recognition receptors (PRRs)) their environment and detect pathogen-associated molecular patterns (PAMPs) [9]. Once they capture antigens, they migrate to lymphoid organs where they present them to T lymphocytes. It has also been shown that DCs participate in the modulation of the immune response towards a Th1 or Th2 response, in the regulation of cytotoxic T lymphocytes, and in immunological tolerance through the production of different cytokines [10,11,12,13,14,15].

## 2. Dentritic Cell Ontogeny

### 2.1. Mouse Dendritic Cells

**Figure 1:**
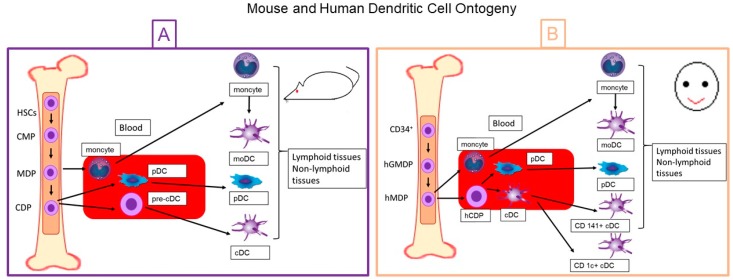
Mouse and human dendritic cell ontogeny. Schematic representation of (**A**) mouse and (**B**) human dendritic cell ontogeny. DC: dendritic cell; HSCs: hematopoietic stem cells; CMP: common myeloid progenitors; MDP: monocyte and dendritic cell progenitor; CDP: common dendritic cell progenitor; pDC: plasmacytoid DCs; pre-cDC: precursors of conventional DCs; moDC: monocyte-derived DC; pDC: plasmacytoid DC; cDC: conventional DC; hGMDP: human granulocyte-monocyte-DC progenitor; hMDP: human monocyte-dendritic progenitors; hCDP: human CDP.

The ontogeny and development of DCs has been more profoundly analyzed in the murine model as compared to the human model. It has been established that in the early stages of mouse embryonic life, cell differentiation begins, which will give rise to DCs. Common myeloid progenitors (CMPs) are generated from bone marrow-resident hematopoietic stem cells (HSCs). At the same time, CMPs give rise to MΦ and DC progenitors (DCPs), which present the phenotypic markers Lin-CXCR1, CD11b-cKit, and CD135 [11,15,16,17,18]. Dendritic cell progenitors directly originate plasmacytoid DCs (pDCs) and precursors of conventional DCs (pre-cDCs), which leave the bone marrow to the bloodstream to later lodge in the tissues in which they develop and differentiate into DCs (Figure 1) [17,19,20,21,22,23]. During the whole process of differentiation of mouse DCs, FMS-like tyrosine kinase 3 receptor ligand (Flt3L) is required, which binds to the cellular receptor R-Flt3L (CD135) [24,25,26]. Also, growth factors such as macrophage-colony stimulating factor (M-CSF) and granulocyte macrophage colony stimulating factor (GM-CSF) participate in the development of progenitor cells, but not in the maturation of DCs [27].

### 2.2. Human Dendritic Cells

**Figure 2:**
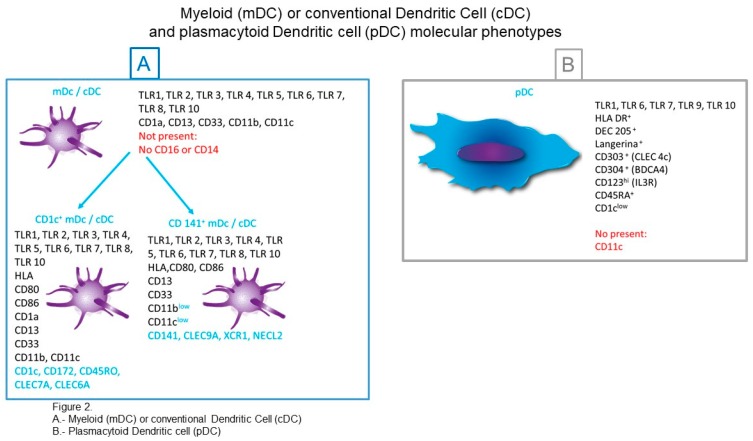
Myeloid or conventional dendritic cells and plasmacytoid dendritic cells molecular phenotypes. Schematic summary of (**A**) myeloid or conventional dendritic cells and (**B**) plasmacytoid dendritic cells molecular phenotypes.

Regarding the origin of human DCs, for a long time there have been several difficulties in establishing its ontogenetic pattern. However, recent success in establishing cultures of CD34^+^ HSCs has provided important information regarding the origin of HDCs. CD34^+^ HSCs give rise to the progenitors of granulocytes, monocytes, and human DC (hGMDPs), which at the same time originate the progenitors of monocytes and human DC (hMDPs) (Figure 2), and monocytes and the common progenitor of human DC (hPDCs). Common progenitor of human DCs, unlike granulocyte and macrophage progenitor (GMP), are not only found in umbilical cord blood and bone marrow but are also located in peripheral blood and lymphoid tissues (Figure 2). Starting from hPDCs, different types of human DCs are generated [28]. These cells are characterized for having an elevated expression of MHC-II, but typically lack the lineage markers CD3 (T cells), CD19/20 (B cells), and CD56 (NK cells). For this reason, DCs have been traditionally referred to as HLA-DR^+^ lineage^−^ cells. Furthermore, to exclude monocytes, which share several surface molecules with DCs, other markers such as CD14 have been used [29].

## 3. Dendritic Cell Subpopulations

There are two main subtypes of DCs: conventional DCs (cDCs), also called myeloid DCs (mDCs) and plasmacytoid DCs (pDCs) [15].

### 3.1. cDCs (Conventional Dendritic Cells) or mDCs (Myeloid Dendritic Cells)

Conventional DCs are derived from pre-cDCs that are produced in the bone marrow. They migrate into the blood and then seed various tissues. Their differentiation in vitro requires GM-CSF and Flt3L and are characterized for the expression of CD1a, CD11c, CD13, CD33, but do not express CD14 or CD16 [30]. Recently, CD26 has been added as a cDC marker [31]. Conventional DCs are also characterized by the expression of different toll-like receptors (TLRs) such as TLR1-TLR8 and TLR10 (Figure 2). According to the expression of surface markers, cDCs are divided into two groups: CD1c^+^ mDC and CD141^+^ mDC [30]. Both subtypes of cDCs display unique gene expression profiles, suggesting specialized functions. In particular, it has been shown that CD1c^+^ DCs express TLR-4, while CD141^+^ do not, which differentiate them in their capacity to respond to *Escherichia coli.* Also, CD1c^+^DCs produce low levels of tumor necrosis factor (TNF), Interleukin (IL)-6, and IL-12 and high levels of IL-10 and regulatory molecules such as indoleamine-2,3-dioxygenase (IDO) and soluble CD25. Moreover, *E. coli*-activated CD1c^+^ DCs have the capacity to suppress T-cell proliferation in an IL-10-dependent manner [32].

Other authors have classified cDCs into cDC1 and cDC2 and several transcription factors have been shown to be required for their development and/or function such as IRF-8, BATF3 and ID2 for cCD1 and IRF4 and ZEB2 for cDC2 [33].

#### 3.1.1. CD1c^+^ cDCs

The percentage of CD1c^+^ cDCs cells present in blood, non-lymphoid tissues, and lymphoid tissues is higher in comparison to that of CD141^+^ cDCs [34]. CD1c^+^ cDCs are characterized for the expression of CD1a, CD11b, CD11c, CD13, CD33, CD172, and CD45RO [35]. Also, tissue CD1c^+^ cDCs express CD80^+^, CD83^+^, CD86^+^, and CD40^+^, which indicates a more active phenotype as compared to that of CD1c^+^ cDCs blood cells [36,37,38]. In addition to CD1c, CD1c^+^ cDCs express CD1a, which is shared with Langerhans cells (LCs); however, the expression of CD1a in LCs is greater [35,36,38,39]. The expression of CD1a and CD1c gives these cells the ability to present glycolipid antigens such as those present in *Mycobacterium tuberculosis* to naive T cells [40]. Other important molecules expressed by CD1c^+^ cDC are the CD13 aminopeptidase that inhibits receptor-mediated antigen uptake and thereby regulates DCs cross-presentation and cell responses [41]. Also, CD13 participates in phagocytic processes in DCs and MΦ [42]. CD33 is a surface marker of CD1c^+^ cDC and is a member of the sialic acid-binding immunoglobulin-like lectin (SIGLEC) family. CD172^+^ (Signal regulatory protein or SIRPα) interacts with a transmembrane protein expressed in most cells known as CD47 or “don’t eat me” signal, the CD172-CD47 interaction produces the inhibition of own cell phagocytosis. The presence of CD172 allows CD1c^+^ cDCs to regulate its phagocytic activity [43]. CD1c^+^ cDCs also express CLRs (C-type lectin receptors) such as of Dectin-1 (CLEC (C-type lectin) 7A) and Dectin-2 (CLEC6A) that suggests the ability of these cells to recognize fungal antigens. The expression of TLRs (1–8) confers CD1c^+^ cDCs the capacity to respond well to lipopolysaccharide, flagellin, and double-stranded RNA [44] and, in response, these cells produce IL-12 [45]. When skin CD1c^+^ cDCs are stimulated, they secrete TNF-α, IL-8, IL-10, and IL-23 [46,47]. On the other hand, the stimulation of these cells with TLR7/TLR8 agonists does not induce the production of IL-12 as has been demonstrated with blood CD1c^+^ cDCs [48]. Also, CD1c^+^ DCs produce high levels of IL-10. Therefore, it is recognized that CD1c^+^ cDCs have plasticity to collaborate in the response of both Th1 and Th17 [45].

#### 3.1.2. CD141^+^ cDCs (Conventional Dendritic Cells)

CD141^+^ cDCs are resident cells of lymph nodes, tonsils, spleen, and bone marrow [49] as well as of non-lymphoid tissues such as skin, lung, and liver [46]. CD141^+^ cDCs express less CD11b and CD11c as compared to CD1c^+^ cDCs [46]. These cells possess the ability to capture dead or necrotic cells by means of CLEC9A, a type V CLR that functions as an activation receptor [50,51]. They also express nectin-like protein 2 (Necl2) [52] and chemokine receptor XCR1 [53]. These cells can sense viral nucleic acids by means of TLR3 and TLR8 [46,51,54]. CD141^+^ cDCs participate in a very important manner in the presentation of exogenous antigens through MHC-I molecules for the initiation of CD8^+^ T cell responses, an event known as cross-presentation [46,51,54].

### 3.2. pDCs (Plasmacytoid DCs)

The name of these cells derives from their appearance similar to plasma cells and are characterized for the production of high amounts of type 1 interferons to the recognition of active or inactivated viruses or by contact with DNA through TLR7 and TLR9 [55]. In addition to these TLRs, they also express TLR1, TLR6, and TLR10. Plasmacytoid DC populations are composed of transcriptionally and functionally heterogeneous cellular subsets with distinct hematopoietic precursor origin. Whereas cDCs originate mostly from a common dendritic cell progenitor (CDP), pDCs have been shown to develop from both CDPs and common lymphoid progenitors. From this last, pDCs develop predominantly from IL-7R^+^ lymphoid progenitor cells, are characterized for high expression of the transcription factor IRF8, and for their in vitro differentiation they require IL-3, but not GM-CSF. Both mature pDC subsets are able to secrete type 1 interferons, but only myeloid-derived pDCs share with cDCs their ability to process and present antigen. The molecule CD123 is the receptor of IL-3, cytokine that participates in the development and proliferation of pDCs [56].

Of the total DCs present in blood, pDCs make up about 50% and of the total blood mononuclear cells, pDCs constitute 1% [57]. In steady state, it is unlikely to find these cells in non-lymphoid organs and are found only in blood and lymphoid organs. Plasmacytoid DCs are practically absent in healthy tissue; however, during inflammation they are rapidly recruited, reaching a greater number in tissues [38,46]. Plasmacytoid DCs lack myeloid markers such as CD11c, CD11b, CD13 and CD33 but express CD45RA, variable CD2 and CD7. Fully differentiated murine pDCs express a unique combination of surface markers including CD11c, B220, Ly6C/G, and Ly49Q [58]. On the other hand, some markers such as CD303 (CLEC4C: BDCA (blood dendritic cell antigens)-2), CD304 (neuropilin: BDCA-4), CD123 (IL-3R) and CD1c (BDAC-1) are unique to humans [59,60,61] (Figure 2). CD303 is involved in ligand internalization, processing and presentation, as well as in inhibition of interferon (IFNα/β)-synthesis in pDCs. On the other hand, CD304 participates in cell survival, migration, and attraction [62]. 

Plasmacytoid DCs can have immunogenic or tolerogenic functions depending on different factors such as their ability to produce inflammatory cytokines [63]. In relation to its immunogenic functions, pDCs participate in the induction of a Th2 type immune response by the production of IL-4 and [30] and also it has been observed that they can interact with NK cells in viral infections [7]. On the other hand, pDCs can elicit suppressive responses as has been demonstrated through their capacity to induce suppressive Tregs through IDO expression. It has been shown that naive pDCs express high amounts of IDO in the lymph nodes through a cross-talk with Foxp3 Tregs. The expression of IDO permits pDCs to confer suppressive functions to Tregs in the context of experimental autoimmune encephalomyelitis (EAE) [64]. Also, it has been shown that that the treatment of human pDCs with all three classes (type A, B, and C) of CpG ODN (oligodeoxynucleotides) prime naïve CD4^+^CD25^+^ T cells to differentiate into CD4^+^CD25^+^ Tregs characterized as Foxp3^+^ IL-10-producing immunosuppressive T cells with strong Ag-nonspecific immunosuppressive effects on naive CD4^+^T cell proliferation [65].

### 3.3. Dendritic Cells That Respond to Specific Microorganisms

#### 3.3.1. TNF-α and iNOS Producing Dendritic Cells (Tip-DCs) and Myeloid-Derived Suppressor Cells (MDSC)

It has been shown that some populations of DCs develop as a specific response to some microorganisms. Among these are the TNF-α and iNOS producing dendritic cells (Tip-DCs) and the myeloid-derived suppressor cells (MDSCs). Mouse Tip-DCs have the phenotype CD11b^int^, CD11c^int^, Gr-1^+^, DEC-205^−^, CD14^−^, F4/80; however, in humans, it has been difficult to establish a characteristic phenotype. Chong et al., 2011 succeeded in differentiating monocytes into Th1 Tip-DCs, which are characterized by the high expression of co-stimulatory molecules, TLR2, 3 and 4, MHC-I and II, DC-SIGN (Dendritic Cell-Specific Intercellular adhesion molecule-3-Grabbing Non-integrin) and the classical DC maturation marker, CD83 [66]. On the other hand, in 2013 Wilsmann-Theis et al. managed to develop a new model for the study of human Tip-DCs and provided the first evidence of their pro-inflammatory capacity. The phenotype of these cells was defined as CD11c^+^, CD86^+^, and CD40^+^, while lacking CD1a, CD1c, or CD207/Langerin. Tip-DCs produce TNF and inducible nitric oxide synthase (iNOS)/nitric oxide (NO) before infection by *Listeria monocytogenes*, which results in an effective mechanism against infection; however, in some cases, this response has been related to tissue damage [67]. It has also been described that in the early stages of infection by *Leishmania* the inflammatory environment produced is ideal to stimulate the differentiation of monocytes towards Tip-DCs.

MDSCs are a heterogeneous population whose phenotype in humans is Lin^−^ HLA DR^−^ CD33^+^ or CD11b^+^ CD14^−^ CD33^+^. In mice, the CD11b^+^ Gr1^+^ cell population consists of two large families, granulocytic MDSCs (CD11b^+^, Ly6G^+^, Ly6C^low^) and monocytic MDSCs (CD11b^+^, Ly6G^+^, Ly6C^high^). These cells have a regulatory role in the immune response, which limits tissue damage [68,69].

### 3.4. Dendritic Cells CD14^+^

CD14^+^ DCs are characterized by the presence of CD14^+^, which suggests that they probably originate from monocytes with which they share more similarities than with cDCs CD11c^+^ and CD141^+^ cells [30]. Since the discovery of moDCs, they have been described as CD14^low/negative^ and CD14 is mainly considered a monocytic marker. However, CD14 DCs do exist in vivo in the skin. In 1993, Nestle et al. isolated from skin cultures a type of cells expressing CD14^+^, these cells were identified as a third subtype of CD11c^+^ cDCs (Figure 2). In order to contrast them with epidermal LCs, these cells were termed “interstitial-type” or “dermal-type” DCs; however, due to the ambiguity of these names with respect to the main interstitial cell population CD1c^+^ DCs, they fell in disuse. Subsequent studies identified this same type of cells in both lymphoid tissue and in various non-lymphoid tissues [70,71,72]. CD14^+^ DCs express CD11c^hi^ and HLA^hi^, but also express other markers that result in an intermediate phenotype between DCs and monocyte/macrophages. The phenotype of CD14^+^ DC is CD14^+^, CD11c^hi^, HLA^hi^, CD163, CD11b, CX3CR1, FXIIIa, CD209 (DC-SIGN), CCR7^−^, CD80^low^, CD86^low^. These cells also express TLRs 1–9 [35,36] and lack markers typical of the other cDCs, such as CD1c or CD141 [35,39,72]. After being stimulated, CD14^+^ DCs secrete IL-1β, IL-6, IL-8, and IL-10 [72]. Because CD14^+^ DCs lack CC chemokine receptor 7 (CCR7), it is unknown whether or not these cells have the ability to migrate to lymph nodes; however, from lymph node samples it has been possible to isolate CD14^+^ and CD209^+^ cells, so it is proposed that they could be cells that migrated from blood [49,73]. CD14^+^ DCs participate in various processes of immunological activity. It has been described that play an important role in the formation of follicular helper T cells [72], induces antibody-secreting B cell differentiation [74] and in vitro has been shown to induce regulatory T cells [75]. Recently, resident CD141^+^ CD14^+^ regulatory DCs have been identified in human skin. In particular, a new population of regulatory DCs was described in hepatocellular carcinoma (HCC) patients, with a unique phenotype of CD141CD11b^high^CTLA-4, representing approximately 13% of PBMCs. These CD141^+^DCs suppress CD4 T-cell response by CTLA-4-dependent IL-10 and IDO production [76].

### 3.5. Dendritic Cells Derived from Monocytes (moDC)

Dendritic cells originate mainly from precursors present in the bone marrow; however, some can be differentiated from other cells, as is the case of DCs derived from monocytes. In humans, there are three types of monocytes: the classical (CD14^+^, CD16^−^), the intermediate (CD14^+^, CD16^+^) and the non-classical ones (CD14^low^, CD16^+^). Currently, it has not been defined exactly from which subtype of monocytes the moDCs are derived in vivo [29]. According to transcriptomic analyzes, it has been suggested that in humans, skin CD14^+^ DCs, as well as DCs CD103^−^ CD172a^+^ of intestine are related to monocytes [70,77] and therefore, these are considered authentic moDC. On the other hand, cells inflammatory tissues that express CD11c, CD1a, and CD14 are most likely derived from monocytes and therefore are considered moDCs [78,79].

## 4. Dendritic Cell Classification Based on Its Anatomical Location

According to the anatomical location, mDCs and, to a lesser extent, pDCs, are divided into blood DCs, peripheral resident DCs in the non-lymphoid tissues and DCs residing in the secondary lymphoid tissues.

### 4.1. Blood Dendritic Cells

Blood DCs in humans can be pDCs and mDCs (both CD1c^+^ and CD141^+^) [57,62].

### 4.2. Dendritic Cell Migrants and Peripheral Residents in Non-Lymphoid Tissues

These cells are also called non-lymphoid DCs and traffic through the tissues. The subtypes of DCs that fall into this category are CD1c^+^ mDCs, CD141^+^ mDCs, CD14^+^ DCs and very few pDCs [46,49] Among the peripheral resident DCs in the non-lymphoid tissues are DCs associated with skin, which are LCs and the interstitial dermal cells (intDCs). The origin of these two populations is still controversial since some authors suggest that the precursor is of myeloid origin and one of the intermediaries is the monocyte [80]. On the other hand, other authors propose that the origin of LCs and intDCs comes from a fetal parent that also gives rise to the cells of the central nervous system glia [81]. LCs are located in the epidermis and express on the surface CD1a, Langerin and E-cadherin.

Interstitial DCs or dermal dendritic cells are located in the dermis, are motile and express high levels of MHCII present in their cytoplasmic dendritic processes and lack numerous T cell, B cell and NK antigens. Three subsets of dermal dendritic cells have been distinguished. All express factor XIIIa, there is a small population from 10 to 15% that express CD14.

In addition, LCs and intDCs differ in the response to certain stimuli and in the production of cytokines and chemokines. For example, stimulation with CD40L induces the production of IL-10 by intDCs and not from the LCs. On the other hand, the intDCs produce IL-6 and IL-12, which induce the differentiation of B cells into plasma cells that produce immunoglobulin M (IgM) and stimulate Th cells for the production of Th1 type cytokines, favoring this type of response. Langerhans cells stimulate Th cells to secrete IL-4, IL-5, and IL-13, resulting in a Th2-type response [30].

Migratory CDs, during an inflammatory process, travel through the lymphoid system from a tissue to the lymph nodes. In murine models, this process is regulated by the CCR7 receptor. The total maturation of the DCs during migration has been associated with the tolerance to the antigen [7].

### 4.3. Dendritic Cell Residents of Secondary Lymphoid Tissues

Also called lymphoid DCs, these DCs arise in lymph nodes directly from the blood and are non-migratory [46,49]. Populations of both resident DCs and migratory DCs are found in the lymph nodes. The residents are in subcapsular sinuses and when there is migration, they are added in the paracortical zone [7]. In humans, it is difficult to differentiate between migratory DCs and steady state residents. The subtypes of DCs that fall into the category of resident DCs are: CD1c^+^ cDCs, CD141^+^ cDCs and pDC in the steady state and very few CD14^+^ [46,49].

## 5. Dendritic Cell Maturation

After differentiation of cDCs or pDCs, DCs are considered immature DCs, some of which are circulating and migrate to infection sites [82]. Immature DCs are excellent capturers of antigens (Ag) and thus are recognized as crucial sentinel cells that evolve from an immature towards a mature cell capable of migrating to lymphoid nodules and become specialized antigen presenting cells (APC) that prime both naïve CD4^+^ and CD8^+^ T cells. Antigen recognition is carried out through different PRRs and immature DCs have high endocytic and phagocytic capacity and low expression of MHC-II and costimulatory molecules and thus the ability to present antigens is poor. Mature DCs, which have already had contact with the antigen, have low endocytic and phagocytic capacity and high antigen presentation capacity. Mature DCs present morphological changes, express higher amounts of molecules MHC-II, CD40, CD80, CD86, and membrane protein associated with lysosomes (DC-LAMP), a protein associated with antigen presentation. Phagosomal pH in DCs is higher compared to that of MΦs, explaining why antigens remain more stable and can be presented practically intact, thus converting antigens into immunogens [83,84]. Once the APC presents the Ag to the naive CD4^+^ or CD8^+^ T cells, they express molecules such as cytokines, chemokines, costimulatory molecules, and proteases to initiate an immune response [82,85].

## 6. Mechanisms Used by Dendritic Cells for Recognition and Antigen Capture

Immature DCs are recruited to sites of inflammation in tissues where they capture Ag, internalize them and migrate to lymph nodes while they mature. Immature DCs express the chemokines CCR1, CCR2, CCR5, CCR6, and CXCR1 [86,87], thereby facilitating the arrival at the site of inflammation, infection and being able to recognize chemo-attractants, such as MIP-3 alpha/CCL20, RANTES/CCL5 and MIP-1 alpha/CCL3 [88]. Antigen recognition is carried out through different receptors, such as FcγRs, FcεRs, and CRs. These receptors recognize opsonized Ag with antibodies or complement fragments, respectively. Dendritic cells can also recognize antigens through other receptors such as PRRs, which can be found in the plasma membrane and in the endosome, as well as in the cytosol. TLRs and CLRs are examples of these membrane PRRs [81]. Within the CLR families, one of the most studied is the CLECs. Members of this family are C-type lectin receptor 1 (CLEC-1), DC-SIGN (CELC4L), langerin (CLEC4K), DC immunoreceptor (DCIR or CLEC4A), BDCA2 (CELC4C), DC-associated C-type lectin-1 and 2 (Dectins 1 and 2 or CLEC7A/6A), mannose receptor types 1 and 2 (CLEC13D/E) and DEC205 (CLEC13B). This family is classified into two groups, type I CTLRs and type II CTLRs. CLECs recognize conserved sequences of oligosaccharides present on surfaces of microorganisms that differ from those of mammals [89]. The ligand for DCIR are glycosylated molecules [90]; however, for the other molecules, its ligand is unknown [91,92,93]. Another important receptor is the mannose receptor (CD206), which together with FcγR, FcεR, and DCIR participate in an important way in Ag handling by immature DCs. Once immature DCs capture Ag via phagocytosis or receptor-mediated endocytosis, endocytosis occurs. Dendritic cells have the capacity to endocytose and phagocytose different particles and microorganisms, which is why they are considered to be professional phagocytic cells [80,94,95]. After the capture of Ag and endocytosis or phagocytosis, DCs maturation begins and during this process the expression of the first markers is lost and with it the ability to capture antigens. Simultaneously, there is an increase in the capacity of antigenic presentation [96,97]. To initiate the maturation process, changes in the level of expression of certain chemokine and chemokine receptor expression profiles occur in DCs. The expression levels of the chemokine receptors CCR6 and CCR7 increase, which allows them to migrate via blood or lymph to the lymph nodes to present the Ag where them will present the Ag either to a naive CD4^+^ or CD8^+^ T cell [80,94,95].

## 7. Mechanisms Used by Dendritic Cells for Antigenic Presentation

### 7.1. Mechanisms of Antigen Presentation by Dendritic Cells

One of the defining characteristics of DCs is their high capacity for the capture, processing, and presentation of Ag, all of them being important cellular processes for the activation of naïve T cells. However, these properties will depend on multiple factors, including DCs subtype, anatomical location, the Ag accessibility, as well as the effects that the pathogen itself can exert on DCs [98]. In general terms, all subtypes of DCs efficiently present MHC-I and II Ag, in turn, these subtypes express high levels of MHC-I and II molecules, especially when they have finished their maturation process [99]. The peptides presented by the MHC-I molecules have a cytosolic processing by the proteasome, while the MHC-II molecules present peptides degraded in endosomal compartments that carry a processing by cathepsins and other hydrolytic enzymes [100].

### 7.2. Antigen Presentation by MHC-I

Dendritic cells infected by viruses or intracellular bacteria can use viral proteins synthesized endogenously for presentation in MHC-I molecules. Within DCs, the ubiquitin protein will bind to a specific sequence of a certain peptide. The peptide-ubiquitin complex binds to the proteasome present in the cytosol or cell nucleus, where there will be a proteolytic degradation that produces linear peptides, which will be transported to the endoplasmic reticulum (ER) by a carrier dimeric protein found in the ER membrane called transporter associated with Ag processing (TAP). The TAP mediates the active and ATP-dependent transport of peptides towards the RE lumen where it will be subsequently associated with the protein capsin, which has affinity for molecules of the MHC-I [101]. Peptides that enter the ER through TAP are trimmed to the appropriate size to bind to MHC-I by the ER-type 1 associated aminopeptidase (ERAP-1) [102]. Once the peptide has been processed, it will be anchored in the groove formed by the α1 and α2 segments of the α chain of MHC-I, this union is known as “peptide loading”. When the “peptide load” occurs, MHCI-I and tapasin separation occurs and, subsequently, the peptide-MCH-I complex leaves the ER moving through the Golgi apparatus and is transported to the cell surface by exocytic vesicles to finally be recognized by CD8^+^ T cells [103].

### 7.3. Cross-Presentation

Traditionally, it is known that exogenous (extracellular) antigens are presented via MHC-II to CD4^+^ T cells and endogenous (cytosolic) antigens are presented by means of MHC-I to CD8^+^ T cells; however, there are some cases of presentation of antigens that cannot be explained by these two traditional routes. For example, the classic MHC-I presentation pathway requires DCs to be infected; however, some viruses do not infect DCs and still generate a CD8^+^ T cytotoxic cellular response. On the other hand, traditional mechanisms do not explain how some pathogens that are phagocytosed such as *Salmonella* or *Leishmania* can induce a cytotoxic response of CD8^+^ T. Finally, another problem not solved by traditional mechanisms is the fact that the antigens of the vaccine are extracellular and still induce a CD8^+^ T cell response.

The answer to these questions is the ability of DCs to present exogenous antigens through MHC-I. This alternative form of presentation is known as cross-presentation.

After phagocytosis by DCs, Ag can be exported to the cytosol or it can be degraded in the phagosome itself, the cellular pathways through which this antigenic assimilation is carried out they can be divided into cytosolic and vacuolar. Cytosolic pathway is sensitive to proteasome inhibitors [104], suggesting that the proteins enter the cytosol to be subsequently degraded by the proteasome, additional post-proteasome processing is also required by the ER-associated aminopeptidase 1 (ERAP-1) [105] and the endosomal aminopeptidase responsive to insulin (IRAP) [106].

Subsequently, these peptides follow the traditional route of presentation by MHC-I being transported by the ER. Phagosomal syntaxin 4 interacts with SNARE SEC22B present in the intermediate compartment AG-RE (ERGIC) for the recruitment of ER components [107], being TAP the most important, however, the loading of peptides does not occur in the ER, but in endocytic compartments [108]. One of the most important characteristics of this pathway is the peptide loading in the cytosol. Experimental evidence suggests that in vivo there is a predominance in the use of this pathway over the vacuolar pathway [109]. Interestingly, it has been shown that the DC subtype that are more efficient in presentation is the CD8α^+^ present in the spleen [110]. The vacuolar presentation pathway is resistant to proteasome inhibitors and usually independent of TAP, but sensitive to lysosomal proteolysis inhibitors, particularly inhibitors of cathepsin S [111]. Based on this evidence, it has been suggested that both peptide processing and Ag load occur in endocytic compartments unlike the cytosolic pathway.

### 7.4. Antigen Presentation by MHC-II Molecules

As DCs maturation process takes place, changes occur in the expression of surface molecules. Subsequent to the capture and internalization of the Ag, there is an over-expression of the costimulatory molecules CD80 and CD86, and transport peptide-loaded MHC-II complexes to the cell surface [112]. The neo-synthesized MHC-II molecules are found with the ligand-peptide complexes, originated from the proteolysis of Ag endocytosed by DCs in the same compartment, where the invariable chain (Ii) is synthesized [113]. Within the endosomes and phagosomes of CDs, hydrolysis is performed by pH-sensitive proteases producing peptides from endocytosed Ag [114]. The loading of peptides to MHC-II is carried out after the proteolytic processing of Ii to peptide associated with invariable class II chain (CLIP). The non-classical MHC molecule HLA-DM facilitates the exchange of CLIP by Ii [115], which functions as a stabilizer of the MHC-II molecule, allowing them to maintain a peptide receptor configuration. Through these series of processes, there is a dissociation of the MHCII-Ii complex to the MHCII-CLIP complex and finally MHCII-peptide (pMHC-II). Subsequently, pMHC-II will be transported to the cell surface via microtubules by a motor of dynein complexes controlled by the lysosomal interaction protein Rab7 (RILP) and the cholesterol sensor ORP1L [116]. The pMHC-II complex found in the cytoplasmic membrane is recognized by T CD4^+^ cells. Generally, after the formation of pMHC-II, it is transported to lysosomes for direct degradation, although there is another pathway formed by multi-vesicular endosomes (MVB) [117], which are formed from intraluminal vesicles that can capture certain particles of the cell surface. Subsequently, targeting the lysosomes and producing degradation of these particles, although as an alternative these same vesicles can exit to the cell surface and be released as exosomes to the extracellular space, which may contribute to the activation of CD4^+^ T cells [118].

## 8. Dendritic Cells and Mucosal Immunity

**Figure 3:**
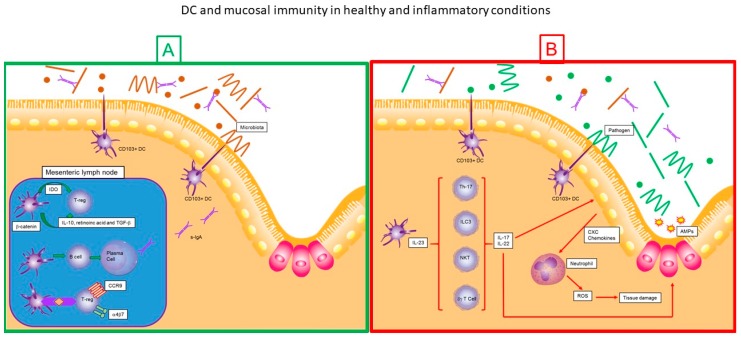
Dendritic cells and mucosal immunity in healthy and inflammatory conditions. Schematic representation of the interaction between gut microbiota, dendritic cells and inflammatory response in gut mucosa in a healthy state (**A**), and gut mucosa in dysbiotic–inflammatory state (**B**). α4β7: α4β7 Integrin, AMPs: antimicrobial peptides, CCR9: C-C chemokine receptor type 9, CXC: CXC Chemokines family, DC: Dendritic Cell, IDO: Indoleamine-pyrrole 2,3-dioxygenase, IL-10: Interleukin 10, IL-17: Interleukin 17, IL-22: Interleukin 22, IL-23: Interleukin 23, ROS: Reactive oxygen species, s-IgA: Secretory Immunoglobulin A, TGF-β: Transforming growth factor beta, T-reg: Regulatory T cells.

The mucosal immune system (MIS) is the most extensive immune organ of the human body and is found covering the intestine, skin, oral and nasal cavities, as well as the vaginal canal. Based on its anatomical and functional properties and for its greater understanding, two zones are described in the MIS, the inductive and the effector. The inductive zone (also known as mucosa-associated lymphoid tissue (MALT)) include gut-associated lymphoid tissues (GALT), nasopharyngeal-associated lymphoid tissue (NALT), and lymphoid sites. The effector zone includes secretory glandular tissue and the lamina propria of the gastrointestinal, upper respiratory and reproductive tissues. Particularly at the intestinal level, in the lamina propria (LP), the DCs recognize the bacteria of the intestinal microbiota and its metabolites as well as food antigens that are found in the intestinal lumen. These are transported to mesenteric lymph nodes (MLN) and Peyer’s patches. To maintain a healthy homeostatic balance at the intestinal level, immunological tolerance to these antigens must be generated, as well as anti-inflammatory conditions (Figure 3). In humans, two subtypes of CD103^+^ (αE integrin) DCs, CD103^+^ CD11b^+^ and CD103^+^ CD11b^−^DCs have been recognized; but until now it is unknown if there is any specific correlation with their participation in tolerance or inflammation. CD103^+^ DCs subset express IDO, which is an enzyme that promotes the development of Tregs. Once the CD103^+^ DCs has captured the antigen, it presents it to Tregs, promoting the secretion of IL-10 as an immunosuppressive and anti-inflammatory mechanism [119]. Dendritic cells that detect bacterial antigens of the intestinal microbiota by means of TLRs and other receptors activate the signaling pathway of β-catenin, inducing the expression of IL-10, retinoic acid, and TGF-β that will further activate Tregs [120].

On the other hand, DCs activate B cells to be transformed into plasma cells that produce immunoglobulin A (IgA). Immunoglobulin A binds Ag from the intestinal microbiota and food, thus limiting its passage to the bloodstream and preventing an immune response against intestinal microbiota or food [121]. It has been demonstrated in the murine model that a characteristic pattern of these CD103^+^ DCs associated with tolerance is the ability to induce the Tregs to express gut-homing markers α4β7 and CCR9. These gut-homing markers allow Tregs to localize immune responses to specific tissues [122,123,124]. The intestinal homeostatic balance can be broken due to an inflammatory process. Irritable bowel syndrome (IBS) and inflammatory bowel disease (IBD) are among the intestinal pathologies associated with inflammation. Inflammatory bowel disease is composed of a group of pathological entities characterized by inflammation of the small intestine and colon. The two main diseases belonging to IBD are ulcerative colitis and Crohn’s disease. The causes that lead to the development of IBD are unknown; however, it has been proposed that its origin be multifactorial, where the genetic predisposition of the patient, nutrition and eating habits, as well as the alteration of the intestinal microbiota balance; The interaction of these factors will influence the pathological status of uncontrolled immune-mediated inflammatory response. By means of rRNA sequencing, in 2007 Frank et al. demonstrated that the bacterial population in patients with IBD had an anomalous distribution, found predominance of the phyla Actinobacter and Proteobacter and the depletion of the phyla Firmicutes and Bacteroidetes, the latter frequently found in healthy patients. This alteration of the population distribution of phyla of the normal microbiota is called dysbiosis [125]. In 2012, Morgan et al. reported that the dysbiosis observed in IBD produced an alteration of the metabolism that leads to oxidative stress and disturbed nutrient availability during tissue damage [126]. In 2014, Gevers et al. demonstrated that antibiotic use amplifies the microbial dysbiosis associated with DCs [127]. There is evidence that in inflammatory bowel disease there is an alteration in lymphocyte trafficking with enhanced lymphocyte expression of gut-homing molecule α4β7; however, the causes that trigger these events are unknown [128,129,130,131]. On the other hand, after activation by pathogenic microorganisms, both MΦ and DCs produce IL-23 and thus activate Th17 cells, T cells, NK cells, natural killer T (NKT) cells and group 3 innate lymphoid cells (ILC3s) The activation of these cells provokes the secretion of IL-17 and IL-22 that stimulate the intestinal epithelium to produce antimicrobial peptides (AMPs) and the secretion of CXC-chemokines that are chemoattractant for neutrophils, which produce and release reactive oxygen species (ROS) [132].

## 9. Conclusions

Dendritic cells comprise a heterogeneous cell population that are considered as sentinel cells of the immune system because they possess the ability to initiate and direct the immune response. They are able to take antigens from their environment, capture, process, and present them to naive T lymphocytes. To carry out these tasks, they must also be able to migrate to the site of infection and inflammation and subsequently migrate from there to lymphoid tissue where they will present the antigen and secrete cytokines to influence or participate in the immune response. Although their discovery dates back to 1868, it is not until recent years that their study has become more important in the light of the search for new therapies and their use as models for vaccines. Another area in which it has gained great importance is in the study of diseases associated with inflammatory mucosal processes such as inflammatory bowel disease and its interaction with the microbiota and mucosal DCs in the intestine. In the case of vaccine development using DCs, these trials have focused on cancer treatment. This therapy is based on the principle that neoplastic processes are pathologies associated with abnormal tissue growth and it is believed that this event may occur when the immune system is “neglected” or unable to detect and destroy or limit the affected tissue. Based on the above, the development of cancer vaccines based on the use of ex vivo “trained” DCs has been proposed. These DCs are obtained from monocytes or CD34^+^ precursors and subsequently are activated with tumor Ags that will be presented to T cells in order to produce an antitumor response [133].

## List of Abbreviations

DCs	dendritic cells
Mφ	macrophages
MHC	major histocompatibility complex
Alpha X	complement component 3 receptor 4 subunit
PRRs	pattern recognition receptors
PAMPs	pathogen-associated molecular patterns
HSCs	hematopoietic stem cells
CMPs	common myeloid progenitors
DCPs	dendritic cell progenitors
pDCs	plasmacytoid dendritic cells
pre-cDC	precursos of conventional dendritic cells
Flt3L	FMS-like tyrosine kinase 3 receptor ligand
M-CSF	macrophage-colony stimulating factor
GM-CSF	granulocyte macrophage colony stimulating factor
hGMDP	progenitor of granulocytes, monocytes and human dendritic cells
hMDP	progenitor of dendritic cells and human monocytes
hPDC	common progenitor of human dendritic cells
GMP	granulocyte and macrophage progenitor
cDCs	conventional dendritic cells
mDCs	myeloid dendritic cells
pDCs	plasmacytoid dendritic cells
TLRs	toll-like receptors
EpCAM	langerin and epithelial cell adhesion molecule
SIGLEC	sialic acid-binding immunoglobulin-like lectin
SIRPα	Signal regulatory protein
TNF-α	necrosis tumor factor-a
IL	Interleukin
CLEC	C-type lectin-like receptor
CTLR	c-type lectin-like receptor
Necl2	nectin-like protein 2
IFN	Interferon
iNOS	inducible nitric oxide synthase
NO	nitric oxide
CCR7	CC chemokine receptor 7
intDCs	interstitial dermal cells
Ig	immunoglobulin
Ag	antigens
APC	antigen presenting cell
DC-LAMP	membrane protein associated with lysosomes
FcγR	receptor of the Fc region of IgG
FcεR	receptor of the Fc region of IgE
CR	complement receptor
DCIR	dendritic cell immunoreceptor
ER	endoplasmic reticulum
TAP	transporter associated with antigen processing
ERAP-1	endoplasmic reticulum-type 1 associated aminopeptidase
IRAP	endosomal aminopeptidase responsive to insulin
ERGIC	intermediate compartment AGRE
Li	invariable chain
CLIP	invariable class II chain
pMHCII	major histocompatibility complex II-peptide
RILP	lysosomal interaction protein Rab7
MVB	multivesicular endosomes
MIS	mucosal immune system
MALT	mucosa-associated lymphoid tissue
GALT	gut-associated lymphoid tissue
NALT	nasopharyngeal-associated lymphoid tissue
MLN	mesenteric lymph nodes
IDO	indoleamine 2,3-dioxygenase
T-reg	regulatory T lymphocytes
IBS	irritable bowel syndrome
IBD	irritable bowel disease
TH17	T helper 17
NKT	natural killer T cells
ILC3s	3 innate lymphoid cells
AMPs	antimicrobial peptides
ROS	reactive oxygen species

## References

[1] Breathnach A.S. (1963). The distribution of Langerhans cells within the human hair follicle, and some observations on its staining properties with gold chloride. J. Anat..

[2] Steinman R.M., Cohn Z.A. (1973). Identification of a novel cell type in peripheral lymphoid organs of mice. I. Morphology, quantitation, tissue distribution. J. Exp. Med..

[3] Steinman R.M., Witmer M.D. (1978). Lymphoid dendritic cells are potent stimulators of the primary mixed leukocyte reaction in mice. Proc. Natl. Acad. Sci. USA.

[4] Steinman R.M. (2004). Dendritic cells: From the fabric of immunology. Clin. Investig. Med..

[5] Nussenzweig M.C., Steinman R.M., Unkeless J.C., Witmer M.D., Gutchinov B., Cohn Z.A. (1981). Studies of the cell surface of mouse dendritic cells and other leukocytes. J. Exp. Med..

[6] Nussenzweig M.C., Steinman R.M., Witmer M.D., Gutchinov B. (1982). A monoclonal antibody specific for mouse dendritic cells. Proc. Natl. Acad. Sci. USA.

[7] Haniffa M., Collin M., Ginhoux F. (2013). Ontogeny and Functional Specialization of Dendritic Cells in Human and Mouse. Adv. Immunol..

[8] Mildner A., Jung S. (2014). Development and function of dendritic cell subsets. Immunity.

[9] Schmid M.A.W. (2012). Characteristics of “Tip-DCs and MDSCs” and their potential role in leishmaniasis. Front. Microbiol..

[10] Shortman K., Liu Y.J. (2002). Mouse and human dendritic cell subtypes. Nat. Rev. Immunol..

[11] Shortman K., Naik S.H. (2007). Steady-state and inflammatory dendritic-cell development. Nat. Rev. Immunol..

[12] Shortman K., Lahoud M.H., Caminschi I. (2009). Improving vaccines by targeting antigens to dendritic cells. Exp. Mol. Med..

[13] Heath W.R., Carbone F.R. (2009). Dendritic cell subsets in primary and secondary T cell responses at body surfaces. Nat. Immunol..

[14] Steinman R.M., Idoyaga J. (2010). Features of the dendritic cell lineage. Immunol. Rev..

[15] Merad M., Sathe P., Helft J., Miller J., Mortha A. (2013). The dendritic cell lineage: Ontogeny and function of dendritic cells and their subsets in the steady state and the inflamed setting. Ann. Rev. Immunol..

[16] Fogg D.K., Sibon C., Miled C., Jung S., Aucouturier P., Littman D.R., Cumano A., Geissmann F. (2006). A clonogenic bone marrow progenitor specific for macrophages and dendritic cells. Science.

[17] Liu K., Victora G.D., Schwickert T.A., Guermonprez P., Meredith M.M., Yao K., Randolph G.J., Rudensky A.Y., Nussenzweig M. (2009). In vivo analysis of dendritic cell development and homeostasis. Science.

[18] Geissmann F., Manz M.G., Jung S., Sieweke M.H., Merad M., Ley K. (2010). Development of monocytes, macrophages, and dendritic cells. Science.

[19] Naik S.H., Sathe P., Park H.-Y., Metcalf D., Proietto A.I., Dakic A., Carotta S., O’Keeffe M., Bahlo M., Papenfuss A. (2007). Development of plasmacytoid and conventional dendritic cell subtypes from single precursor cells derived in vitro and in vivo. Nat. Immunol..

[20] Onai N., Obata-Onai A., Schmid M.A., Manz M.G. (2007). Flt3 in regulation of type I interferon-producing cell and dendritic cell development. Ann. N. Y. Acad. Sci..

[21] Onai N., Obata-Onai A., Schmid M.A., Ohteki T., Jarrossay D., Manz M.G. (2007). Identification of clonogenic common Flt3 M-CSFR plasmacytoid and conventional dendritic cell progenitors in mouse bone marrow. Nat. Immunol..

[22] Ginhoux F., Liu K., Helft J., Bogunovic M., Greter M., Hashimoto D., Price J., Yin N., Bromberg J., Lira S. (2009). The origin and development of nonlymphoid tissue CD103 DCs. J. Exp. Med..

[23] Onai N., Kurabayashi K., Hosoi-Amaike M., Toyama-Sorimachi N., Matsushima K., Inaba K., Ohteki T. (2013). A clonogenic progenitor with prominent plasmacytoid dendritic cell developmental potential. Immunity.

[24] McKenna H.J., Stocking K.L., Miller R.E., Brasel K., De Smedt T., Maraskovsky E., Maliszewski C.R., Lynch D.H., Smith J., Pulendran B. (2000). Mice lacking flt3 ligand have deficient hematopoiesis affecting hematopoietic progenitor cells, dendritic cells, and natural killer cells. Blood.

[25] Karsunky H., Merad M., Cozzio A., Weissman I.L., Manz M.G. (2003). Flt3 ligand regulates dendritic cell development from Flt3 lymphoid and myeloid-committed progenitors to Flt3 dendritic cells in vivo. J. Exp. Med..

[26] Waskow C., Liu K., Darrasse-Jèze G., Guermonprez P., Ginhoux F., Merad M., Shengelia T., Yao K., Nussenzweig M. (2008). The receptor tyrosine kinase Flt3 is required for dendritic cell development in peripheral lymphoid tissues. Nat. Immunol..

[27] Kingston D., Schmid M.A., Onai N., Obata-Onai A., Baumjohann D., Manz M.G. (2009). The concerted action of GM-CSF and Flt3-ligand on in vivo dendritic cell homeostasis. Blood.

[28] Lee J., Breton G., Oliveira T.Y.K., Zhou Y.J., Aljoufi A., Puhr S., Cameron M.J., Sékaly R.P., Nussenzweig M.C., Liu K. (2015). Restricted dendritic cell and monocyte progenitors in human cord blood and bone marrow. J. Exp. Med..

[29] Ziegler-Heitbrock L., Ancuta P., Crowe S., Dalod M., Grau V., Hart D.N., Leenen P.J., Liu Y.J., MacPherson G., Randolph G.J. (2010). Nomenclature of monocytes and dendritic cells in blood. Blood.

[30] Collin M., Mcgovern N., Haniffa M. (2013). Human dendritic cell subsets. Immunology.

[31] Guilliams M., Dutertre C.A., Scott C.L., McGovern N., Sichien D., Chakarov S., Van Gassen S., Chen J., Poidinger M., De Prijck S. (2016). Unsupervised high-dimensional analysis aligns dendritic cells across tissues and species. Immunity.

[32] Kassianos A.J., Hardy M.Y., Ju X., Vijayan D., Ding Y., Vulink A.J., McDonald K.J., Jongbloed S.L., Wadley R.B., Wells C. (2012). Human CD1c (BDCA-1)^+^ myeloid dendritic cells secrete IL-10 and display an immuno-regulatory phenotype and function in response to *Escherichia coli*. Eur. J. Immunol..

[33] Breton G., Lee J., Zhou Y.J., Schreiber J.J., Keler T., Puhr S., Anandasabapathy N., Schlesinger S., Caskey M., Liu K. (2015). Circulating precursors of human CD1c^+^ and CD141^+^ dendritic cells. J. Exp. Med..

[34] O’Keeffe M., Mok W.H., Radford K.J. (2015). Human dendritic cell subsets and function in health and disease. Cell. Mol. Life Sci..

[35] Haniffa M., Ginhoux F., Wang X.N., Bigley V., Abel M., Dimmick I., Bullock S., Grisotto M., Booth T., Taub P. (2009). Differential rates of replacement of human dermal dendritic cells and macrophages during hematopoietic stem cell transplantation. J. Exp. Med..

[36] Angel C.E., George E., Ostrovsky L.L., Dunbar P.R. (2007). Comprehensive analysis of MHC-II expression in healthy human skin. Immunol. Cell Biol..

[37] McLellan A.D., Heiser A., Sorg R.V., Fearnley D.B., Hart D.N. (1998). Dermal dendritic cells associated with T lymphocytes in normal human skin display an activated phenotype. J. Investig. Dermatol..

[38] Zaba L.C., Fuentes-Duculan J., Steinman R.M., Krueger J.G., Lowes M.A. (2007). Normal human dermis contains distinct populations of CD11c^+^BDCA-1^+^ dendritic cells and CD163^+^FXIIIA^+^ macrophages. J. Clin. Investig..

[39] Angel C.E., George E., Brooks A.E., Ostrovsky L.L., Brown T.L., Dunbar P.R. (2006). Cutting edge: CD1a+ antigen-presenting cells in human dermis respond rapidly to CCR7 ligands. J. Immunol..

[40] Van Rhijn I., Ly D., Moody D.B. (2013). CD1a, CD1b, and CD1c in immunity against mycobacteria. Adv. Exp. Med. Biol..

[41] Ghosh M., McAuliffe B., Subramani J., Basu S., Shapiro L.H. (2012). CD13 regulates dendritic cell cross-presentation and T cell responses by inhibiting receptor-mediated antigen uptake. J. Immunol..

[42] Villaseñor-Cardoso M.I., Frausto-Del-Río D.A., Ortega E. (2013). Aminopeptidase N (CD13) is Involved in Phagocytic Processes in Human Dendritic Cells and Macrophages. Biomed. Res. Int..

[43] Gardai S.J., McPhillips K.A., Frasch S.C., Janssen W.J., Starefeldt A., Murphy-Ullrich J.E., Bratton D.L., Oldenborg P.A., Michalak M., Henson P.M. (2005). Cell-surface calreticulin initiates clearance of viable or apoptotic cells through trans-activation of LRP on the phagocyte. Cell.

[44] Van der Aar A.M., Sylva-Steenland R.M., Bos J.D., Kapsenberg M.L., de Jong E.C., Teunissen M.B. (2007). Loss of TLR2, TLR4, and TLR5 on Langerhans cells abolishes bacterial recognition. J. Immunol..

[45] Mittag D., Proietto A.I., Loudovaris T., Mannering S.I., Vremec D., Shortman K., Harrison L.C. (2011). Human dendritic cell subsets from spleen and blood are similar in phenotype and function but modified by donor health status. J. Immunol..

[46] Haniffa M., Shin A., Bigley V., McGovern N., Teo P., See P., Wasan P.S., Wang X.N., Malinarich F., Malleret B. (2012). Human tissues contain CD141^hi^ cross-presenting dendritic cells with functional homology to mouse CD103^+^ nonlymphoid dendritic cells. Immunity.

[47] Morelli A.E., Rubin J.P., Erdos G., Tkacheva O.A., Mathers A.R., Zahorchak A.F., Thomson A.W., Falo L.D., Larregina A.T. (2005). CD4^+^T cell responses elicited by different subsets of human skin migratory dendritic cells. J. Immunol..

[48] Schlitzer A., McGovern N., Teo P., Zelante T., Atarashi K., Low D., Ho A.W., See P., Shin A., Wasan P.S. (2013). IRF4 transcription factor-dependent CD11b^+^ dendritic cells in human and mouse control mucosal IL-17 cytokine responses. Immunity.

[49] Segura E., Valladeau-Guilemond J., Donnadieu M.H., Sastre-Garau X., Soumelis V., Amigorena S. (2012). Characterization of resident and migratory dendritic cells in human lymph nodes. J. Exp. Med..

[50] Sancho D., Joffre O.P., Keller A.M., Rogers N.C., Martinez D., Hernanz-Falcon P., Rosewell I., Reis e Sousa C. (2009). Identification of a dendritic cell receptor that couples sensing of necrosis to immunity. Nature.

[51] Bachem A., Guttler S., Hartung E., Ebstein F., Schaefer M., Tannert A., Salama A., Movassaghi K., Opitz C., Mages H.W. (2010). Superior antigen cross-presentation and XCR1 expression define human CD11c^+^CD141^+^ cells as homologues of mouse CD8^+^ dendritic cells. J. Exp. Med..

[52] Galibert L., Diemer G.S., Liu Z., Johnson R.S., Smith J.L., Walzer T., Comeau M.R., Rauch C.T., Wolfson M.F., Sorensen R.A. (2005). Nectin-like protein 2 defines a subset of T-cell zone dendritic cells and is a ligand for class-I-restricted T-cell-associated molecule. J. Biol. Chem..

[53] Crozat K., Guiton R., Contreras V., Feuillet V., Dutertre C.A., Ventre E., Vu Manh T.P., Baranek T., Storset A.K., Marvel J. (2010). The XC chemokine receptor 1 is a conserved selective marker of mammalian cells homologous to mouse CD8alpha^+^ dendritic cells. J. Exp. Med..

[54] Jongbloed S.L., Kassianos A.J., McDonald K.J., Clark G.J., Ju X., Angel C.E., Chen C.J., Dunbar P.R., Wadley R.B., Jeet V. (2010). Human CD141^+^ (BDCA-3)^+^ dendritic cells (DCs) represent a unique myeloid DC subset that cross-presents necrotic cell antigens. J. Exp. Med..

[55] Liu Y.J. (2005). IPC: Professional type 1 interferon-producing cells and plasmacytoid dendritic cell precursors. Annu. Rev. Immunol..

[56] Lutz M.B. (2004). IL-3 in dendritic cell development and function: A comparison with GM-CSF and IL-4. Immunobiology.

[57] MacDonald K.P., Munster D.J., Clark G.J., Dzionek A., Schmitz J., Hart D.N. (2002). Characterization of human blood dendritic cell subsets. Blood.

[58] Omatsu Y., Iyoda T., Kimura Y., Maki A., Ishimori M., Toyama-Sorimachi N., Inaba K. (2005). Development of Murine Plasmacytoid Dendritic Cells Defined by Increased Expression of an Inhibitory NK Receptor, Ly49Q. J. Immunol..

[59] Reizis B., Bunin A., Ghosh H.S., Lewis K.L., Sisirak V. (2011). Plasmacytoid dendritic cells: Recent progress and open questions. Annu. Rev. Immunol..

[60] Dzionek A., Fuchs A., Schmidt P., Cremer S., Zysk M., Miltenyi S., Buck D.W., Schmitz J. (2000). BDCA-2, BDCA-3, and BDCA-4: Three markers for distinct subsets of dendritic cells in human peripheral blood. J. Immunol..

[61] Mathan T.S., Figdor C.G., Buschow S.I. (2013). Human plasmacytoid dendritic cells: From molecules to intercellular communication network. Front. Immunol..

[62] Dzionek A., Sohma Y., Nagafune J., Cella M., Colonna M., Facchetti F., Günther G., Johnston I., Lanzavecchia A., Nagasaka T. (2001). BDCA-2, a novel plasmacytoid dendritic cell-specific type II C-type lectin, mediates antigen capture and is a potent inhibitor of interferon alpha/beta induction. J. Exp. Med..

[63] Swiecki M., Colonna M. (2015). The multifaceted biology of plasmacytoid dendritic cells. Nat. Rev. Immunol..

[64] Lippens C., Duraes F.V., Dubrot J., Brighouse D., Lacroix M., Irla M., Aubry-Lachainaye J.P., Reith W., Judith N., Mandl J.N. (2016). IDO-orchestrated crosstalk between pDCs and Tregs inhibits autoimmunity. J. Autoimmun..

[65] Moseman E.A., Liang X., Dawson A.J., Panoskaltsis-Mortari A., Krieg A.M., Liu Y.J., Blazar B.R., Chen W. (2004). Human plasmacytoid dendritic cells activated by CpG oligodeoxynucleotides induce the generation of CD4^+^CD25^+^ regulatory T cells. J. Immunol..

[66] Chong S.Z., Wong K.L., Lin G., Yang C.M., Wong S.C., Angeli V., Macary P.A., Kemeny D.M. (2011). Human CD8 T cells drive Th1 responses through the differentiation of TNF/iNOS-producing dendritic cells. Eur. J. Immunol..

[67] Wilsmann-Theis D., Koch S., Mindnich C., Bonness S., Schnautz S., von Bubnoff D., Bieber T. (2013). Generation and functional analysis of human TNF-a/iNOS-producing dendritic cells (Tip-DC). Allergy.

[68] Serbina N.V., Pamer E.G. (2006). Monocyte emigration from bone marrow during bacterial infection requires signals mediated by chemokine receptor CCR2. Nat. Immunol..

[69] Gabrilovich D.I., Nagaraj S. (2009). Myeloid-derived suppressor cells as regulators of the immune system. Nat. Rev. Immunol..

[70] Nestle F.O., Zheng X.G., Thompson C.B., Turka L.A., Nickoloff B.J. (1993). Characterization of dermal dendritic cells obtained from normal human skin reveals phenotypic and functionally distinctive subsets. J. Immunol..

[71] Klechevsky E., Liu M., Morita R., Banchereau R., Thompson-Snipes L., Palucka A.K., Ueno H., Banchereau J. (2009). Understanding human myeloid dendritic cell subsets for the rational design of novel vaccines. Hum. Immunol..

[72] Klechevsky E., Morita R., Liu M., Cao Y., Coquery S., Thompson-Snipes L., Briere F., Chaussabel D., Zurawski G., Palucka A.K. (2008). Functional specializations of human epidermal langerhans cells and CD14^+^ dermal dendritic cells. Immunity.

[73] Angel C.E., Chen C.J., Horlacher O.C., Winkler S., John T., Browning J., MacGregor D., Cebon J., Dunbar P.R. (2009). Distinctive localization of antigen-presenting cells in human lymph nodes. Blood.

[74] Matthews K., Chung N.P.Y., Klasse P.J., Moore J.P., Sanders R.W. (1950). Potent induction of antibody-secreting B-cells by human dermal-derived CD14^+^ dendritic cells triggered by dual Toll-like receptor ligation. J. Immunol..

[75] Chu C.-C., Ali N., Karagiannis P., Di Meglio P., Skowera A., Napolitano L., Barinaga G., Grys K., Sharif-Paghaleh E., Karagiannis S.N. (2012). Resident CD141 (BDCA3)^+^ dendritic cells in human skin produce IL-10 and induce regulatory T cells that suppress skin inflammation. J. Exp. Med..

[76] Han Y., Chen Z., Yang Y., Jiang Z., Gu Y., Liu Y., Lin C., Pan Z., Yu Y., Jiang M. (2014). Human Cd14^+^ CTLA-4^+^ regulatory dendritic cells suppress T-cell response by cytotoxic T-lymphocyte antigen-4-dependent IL-10 and indoleamine-2,3-dioxygenase production in hepatocellular carcinoma. Hepatology.

[77] Watchmaker P.B., Lahl K., Lee M., Baumjohann D., Morton J., Kim S.J., Zeng R., Dent A., Ansel K.M., Diamond B. (2013). Comparative transcriptional and functional profiling defines conserved programs of intestinal DC differentiation in humans and mice. Nat. Immunol..

[78] Guttman-Yassky E., Lowes M.A., Fuentes-Duculan J., Whynot J., Novitskaya I., Cardinale I., Haider A., Khatcherian A., Carucci J.A., Bergman R. (2007). Major differences in inflammatory dendritic cells and their products distinguish atopic dermatitis from psoriasis. J. Allergy Clin. Immunol..

[79] Segura E., Touzot M., Bohineust A., Cappuccio A., Chiocchia G., Hosmalin A., Dalod M., Soumelis V., Amigorena S. (2013). Human inflammatory dendritic cells induce th17 cell differentiation. Immunity.

[80] Banchereau J., Klechevsky E., Schmitt N., Morita R., Palucka K., Ueno H. (2009). Harnessing human dendritic cell subsets to design novel vaccines. Ann. N. Y. Acad. Sci..

[81] Auffray C., Sieweke M.H., Geiss-Mann F. (2009). Blood monocytes: Development, heterogeneity, and relationship with dendritic cells. Annu. Rev. Immunol..

[82] Sato K.K., Fujita S.S. (2007). Dendritic cells: Nature and classification. Allergol. Int. (Jpn. Soc. Allergol.).

[83] Mantegazza A.R., Savina A., Vermeulen M., Pérez L., Geffner J., Hermine O., Rosenzweig S.D., Faure F., Amigorena S. (2008). NADPH oxidase controls phagosomal pH and antigen cross-presentation in human dendritic cells. Blood.

[84] Liu X., Lu L., Yang Z., Palaniyandi S., Zeng R., Gao L.Y., Mosser D.M., Roopenian D.C., Zhu X. (2011). The neonatal FcR-mediated presentation of immune-complexed antigen is associated with endosomal and phagosomal pH and antigen stability in macrophages and dendritic cells. J. Immunol..

[85] Romani N.N., Koide S.S., Crowley M.M., Witmer-Pack M.M., Livingstone A.M.A., Fathman C.G.C., Inaba K., Steinman R.M. (1989). Presentation of exogenous protein antigens by dendritic cells to T cell clones. Intact protein is presented best by immature, epidermal Langerhans cells. J. Exp. Med..

[86] Sallusto F., Palermo B., Lenig D., Miettinen M., Matikainen S., Julkunen I., Forster R., Burgstahler R., Lipp M., Lanzavecchia A. (1999). Distinct patters and kinetics of chemokine production regulate dendritic cell function. Eur. J. Immunol..

[87] Yanagihara S., Komura E., Nagafune J., Watarai H., Yamaguchi Y. (1998). EBI1/CCR7 Is a New Member of Dendritic Cell Chemokine Receptor That Is Up-Regulated upon Maturation. Immunology.

[88] Dieu M.-C., Vanbervliet B., Vicari A., Bridon J.-M., Oldham E., Ait-Yahia S., Briére F., Zlotnik A., Lebecque S., Caux C. (1998). Selective Recruitment of Immature and Mature Dendritic Cells by Distinct Chemokines Expressed in Different Anatomic Sites. J. Exp. Med..

[89] Cunningham A.L., Harman A., Kim M., Nasr N., Lai J. (2013). Immunobiology of dendritic cells and the influence of HIV infection. Adv. Exp. Med. Biol..

[90] Nagae M., Ikeda A., Hanashima S., Kojima T., Matsumoto N., Yamamoto K., Yamaguchi Y. (2016). Crystal structure of human dendritic cell inhibitory receptor C-type lectin domain reveals the binding mode with N-glycan. FEBS Lett..

[91] Bates E.E., Fournier N., Garcia E., Valladeau J., Durand I., Pin J.J., Zurawski S.M., Patel S., Abrams J.S., Lebecque S. (1999). APCs express DCIR, a novel C-type lectin surface receptor containing an immunoreceptor tyrosine-based inhibitory motif. J. Immunol..

[92] Ariizumi K., Shen G.-L., Shikano S., Ritter I.I.I.R., Zukas P., Edelbaum D., Morita A., Takashima A. (2000). Cloning of a Second Dendritic Cell-associated C-type Lectin (Dectin-2) and Its Alternatively Spliced Isoforms. J. Biol. Chem..

[93] Colonna M., Samaridis J., Angman L. (2000). Molecular characterization of two novel C-type lectin-like receptors, one of which is selectively expressed in human dendritic cells. Eur. J. Immunol..

[94] Geijtenbeek T.B., Vliet S.J.V., Engering A., ‘t Hart B.A., Kooyk Y.V. (2004). Self- and nonself recognition by c-type lectins on dendritic cells. Ann. Rev. Immunol..

[95] Nouri-Shirazi M., Banchereau J., Fay J., Palucka K. (2000). Dendritic cell based tumor vaccines. Immunol. Lett..

[96] Regnault A., Lankar D., Lacabanne V., Rodriguez A., Théry C., Rescigno M., Saito T., Verbeek S., Bonnerot C., Ricciardi-Castagnoli P. (1999). Fcgamma receptor-mediated induction of dendritic cell maturation and major histocompatibility complex class I-restricted antigen presentation after immune complex internalization. J. Exp. Med..

[97] Sallusto F.F., Lanzavecchia A.A. (1994). Efficient presentation of soluble antigen by cultured human dendritic cells is maintained by granulocyte/macrophage colony-stimulating factor plus interleukin 4 and downregulated by tumor necrosis factor alpha. J. Exp. Med..

[98] Villadangos J.A., Schnorrer P. (2007). Intrinsic and cooperative antigen-presenting functions of dendritic-cell subsets in vivo. Nat. Rev. Immunol..

[99] Wilson N.S., Villadangos J.A. (2005). Regulation of antigen presentation and cross-presentation in the dendritic cell network: Facts, hypothesis, and immunological implications. Adv Immunol..

[100] Veeraswamy R.K., Cella M., Colonna M., Unanue E.R. (2003). Dendritic Cells Process and Present Antigens Across a Range of Maturation States. J. Immunol..

[101] Zhang Y., Williams D.B. (2006). Assembly of MHC class I molecules within the endoplasmic reticulum. Immunol Res..

[102] Serwold T., González F., Kim J., Jacob R., Shastri N. (2002). ERAAP customizes peptides for MHC class I molecules in the endoplasmic reticulum. Nature.

[103] Van Montfoort N., van der Aa E., Woltman A.M. (2014). Understanding MHC class I presentation of viral antigens by human dendritic cells as a basis for rational design of therapeutic vaccines. Front. Immunol..

[104] Kovacsovics-Banowski M., Rock K.L. (1995). A phagosome-to-cytosol pathway for exogenous antigens presented on MHC class I molecules. Science.

[105] Firat E., Saveanu L., Aichele P., Staeheli P., Huai J., Gaedicke S., Nil A., Besin G., Kanzler B., van Endert P. (2007). The role of endoplasmic reticulum-associated aminopeptidase 1 in immunity to infection and in cross-presentation. J. Immunol..

[106] Saveanu L., Caroll O., Weimershaus M., Guermonprez P., Firat E., Lindo V., Greer F., Davoust J., Kratzer R., Keller S.R. (2009). IRAP identifies an endosomal compartment required for MHC class I cross-presentation. Science.

[107] Joffre O.P., Segura E., Savina A., Amigorena S. (2012). Cross-presentation by dendritic cells. Nat. Rev. Immunol..

[108] Burgdorf S., Schölz C., Kautz A., Tampé R., Kuts C. (2008). Spatial and mechanistic separation of cross-presentation and endogenous antigen presentation. Nat. Immunol..

[109] Rock K.L., Shen L. (2005). Cross-presentation: Underlying mechanisms and role in immune surveillance. Immunol. Rev..

[110] Den Haan J.M., Bevan M.J. (2002). Constitutive versus Activation-dependent Cross-Presentation of Immune Complexes by CD8^+^ and CD8^−^ Dendritic Cells In Vivo. J. Exp. Med..

[111] Shen L., Sigal L.J., Boes M., Rock K.L. (2004). Important role of cathepsin S in generating peptides for TAP-independent MHC class I crosspresentation in vivo. Immunity.

[112] Cella M., Sallusto F., Lanzavecchia A. (1997). Origin, maturation and antigen presenting function of dendritic cells. Curr. Opin. Immunol..

[113] Watts C. (2012). The endosome-lysosome pathway and information generation in the immune system. Biochim. Biophys. Acta.

[114] Ten Broeke T., Wubbolts R., Stoorvogel W. (2013). MHC class II antigen presentation by dendritic cells regulated through endosomal sorting. Cold Spring Harb. Perspect. Biol..

[115] Weber D.A., Evavold B.D., Jensen P.E. (1996). Enhanced dissociation of HLA-DR-bound peptides in the presence of HLA-DM. Science.

[116] Rocha N., Kuijl C., van der Kant R., Janssen L., Houben D., Janssen H., Zwart W., Neefjes J. (2009). Cholesterol sensor ORP1L contacts the ER protein VAP to control Rab7-RILP-p150 Glued and late endosome positioning. J. Cell Biol..

[117] Piper R.C., Katzmann D.J. (2007). Biogenesis and function of multivesicular bodies. Annu. Rev. Cell Dev. Biol..

[118] Théry C., Zitvogel L., Amigorena S. (2002). Exosomes: Composition, biogenesis and function. Nat. Rev. Immunol..

[119] Matteoli G., Mazzini E., Iliev I.D., Mileti E., Fallarino F., Puccetti P., Chieppa M., Rescigno M. (2010). Gut CD103^+^ dendritic cells express indoleamine 2,3-dioxygenase which influences T regulatory/T effector cell balance and oral tolerance induction. Gut.

[120] Manicassamy S., Reizis B., Ravindran R., Nakaya H., Salazar-Gonzalez R.M., Wang Y.C., Pulendran B. (2010). Activation of β-catenin in dendritic cells regulates immunity versus tolerance in the intestine. Science.

[121] Mantis N.J., Forbes S.J. (2010). Secretory IgA: Arresting microbial pathogens at epithelial borders. Immunol. Investig..

[122] Johansson-Lindbom B., Svensson M., Wurbel M.A., Malissen B., Márquez G., Agace W. (2003). Selective generation of gut tropic T cells in gut-associated lymphoid tissue (GALT): Requirement for GALT dendritic cells and adjuvant. J. Exp. Med..

[123] Mora J.R., Bono M.R., Manjunath N., Weninger W., Cavanagh L.L., Rosemblatt M., Von Andrian U.H. (2003). Selective imprinting of gut-homing T cells by Peyer’s patch dendritic cells. Nature.

[124] Stagg A.J., Kamm M.A., Knight S.C. (2002). Intestinal dendritic cells increase T cell expression of alpha4beta7 integrin. Eur. J. Immunol..

[125] Frank D.N., Amand A.L., Feldman R.A., Boedeker E.C., Harpaz N., Pace N.R. (2007). Molecular-phylogenetic characterization of microbial community imbalances in human inflammatory bowel diseases. Proc. Natl. Acad. Sci. USA.

[126] Morgan X.C., Tickle T.L., Sokol H., Gevers D., Devaney K.L., Ward D.V., Reyes J.A., Shah S.A., LeLeiko N., Snapper S.B. (2012). Dysfunction of the intestinal microbiome in inflammatory bowel disease and treatment. Genome Biol..

[127] Gevers D., Kugathasan S., Denson L.A., Vázquez-Baeza Y., Van Treuren W., Ren B., Schwager E., Knights D., Song S.J., Yassour M. (2014). The treatment-naive microbiome in new-onset Crohn’s disease. Cell Host Microbe.

[128] Arihiro S., Ohtani H., Suzuki M., Murata M., Ejima C., Oki M., Kinouchi Y., Fukushima K., Sasaki I., Makamura S. (2002). Differential expression of mucosal addressin cell adhesion molecule-1 (MAdCAM-1) in ulcerative colitis and Crohn’s disease. Pathol. Int..

[129] Briskin M., Winsor-Hines D., Shyjan A., Cochran N., Bloom S., Wilson J., McEvoy L.M., Butcher E.C., Kassam N., Mackay C.R. (1997). Human mucosal addressin cell adhesion molecule-1 is preferentially expressed in intestinal tract and associated lymphoid tissue. Am. J. Pathol..

[130] Hart A.L., Kamm M.A., Knight S.C., Stagg A.J. (2004). Prospective evaluation of intestinal homing memory T cells in ulcerative colitis. Inflamm. Bowel Dis..

[131] Hart A.L., Kamm M.A., Knight S.C., Stagg A.J. (2004). Quantitative and functional characteristics of intestinal-homing memory T cells: Analysis of Crohn’s disease patients and healthy controls. Clin. Exp. Immunol..

[132] Perez-Lopez A., Behnsen J., Nuccio S.P., Raffatellu M. (2016). Mucosal immunity to pathogenic intestinal bacteria. Nat. Rev. Immunol..

[133] Bol K.F., Schreibelt G., Gerritsen W.R., de Vries I.J., Figdor C.G. (2016). Dendritic Cell-Based Immunotherapy: State of the Art and Beyond. Clin. Cancer Res..

